# Dissecting closely linked association signals in combination with the mammalian phenotype database can identify candidate genes in dairy cattle

**DOI:** 10.1186/s12863-019-0717-0

**Published:** 2019-01-29

**Authors:** Zexi Cai, Bernt Guldbrandtsen, Mogens Sandø Lund, Goutam Sahana

**Affiliations:** Center for Quantitative Genetics and Genomics, Department of Molecular Biology and Genetics, Aarhus University, 8830 Tjele, Denmark

**Keywords:** Dairy cattle, Milk traits, GWAS, Closely linked association signals, Candidate genes

## Abstract

**Background:**

Genome-wide association studies (GWAS) have been successfully implemented in cattle research and breeding. However, moving from the associations to identify the causal variants and reveal underlying mechanisms have proven complicated. In dairy cattle populations, we face a challenge due to long-range linkage disequilibrium (LD) arising from close familial relationships in the studied individuals. Long range LD makes it difficult to distinguish if one or multiple quantitative trait loci (QTL) are segregating in a genomic region showing association with a phenotype. We had two objectives in this study: 1) to distinguish between multiple QTL segregating in a genomic region, and 2) use of external information to prioritize candidate genes for a QTL along with the candidate variants.

**Results:**

We observed fixing the lead SNP as a covariate can help to distinguish additional close association signal(s). Thereafter, using the mammalian phenotype database, we successfully found candidate genes, in concordance with previous studies, demonstrating the power of this strategy. Secondly, we used variant annotation information to search for causative variants in our candidate genes. The variant information successfully identified known causal mutations and showed the potential to pinpoint the causative mutation(s) which are located in coding regions.

**Conclusions:**

Our approach can distinguish multiple QTL segregating on the same chromosome in a single analysis without manual input. Moreover, utilizing information from the mammalian phenotype database and variant effect predictor as post-GWAS analysis could benefit in candidate genes and causative mutations finding in cattle. Our study not only identified additional candidate genes for milk traits, but also can serve as a routine method for GWAS in dairy cattle.

**Electronic supplementary material:**

The online version of this article (10.1186/s12863-019-0717-0) contains supplementary material, which is available to authorized users.

## Background

Over the last decade, the development of high density single nucleotide polymorphism (SNP) arrays and next-generation sequencing (NGS) technologies have made genome-wide marker sets available in many organisms [1, 2]. Combining these with phenotypic records on many individuals, genome-wide associate studies (GWAS) present a powerful tool to undercover genetic variants associated with variation in the phenotype [3]. In human, numerous studies successfully identified causal variants for traits such as height [4], bodyweight [5] as well as several complex diseases [6]. However, in livestock, long range linkage disequilibrium typically results in imprecise determination of quantitative trait loci (QTL) locations and the associated genomic regions containing several positional candidate genes. In addition, two or more QTL located close to each other may be misidentified as one QTL. In such situations, additional analyses need to be performed to distinguish multiple QTL located close to each other.

To resolve these issues, we need additional information over and above association statistics. For traits with Mendelian inheritance, techniques such as homozygosity mapping and studies of recombinant haplotypes provide important clues due to the unambiguous association of at least some genotypes with phenotypic differences. For quantitative traits, no such close associations exist. However, genomic information of various types do allow relative prioritization among candidate variants. The challenges are which information to consider post-GWAS and how to combine them with GWAS statistics. Expression quantitative trait loci (eQTL) mapping can help; expression profiles as the dependent trait in a GWAS have identified causal genes in some studies [7]. Nevertheless, eQTL studies are time consuming and expensive. Therefore, alternative approaches to incorporate gene expression data into GWAS are needed. Other sources of additional information like variants’ annotation [8] and evolutionary conservation scores [9] have been used. Unfortunately, these analyses need to be designed on a case-by-case basis [10]. Their implementation is challenging in livestock due to the sparsity of annotation data.

In this study, we used an approach to separate multiple closely linked QTL in dairy cattle by fixing the lead SNP as a covariate. This approach detects QTL chromosome by chromosome, and generates a list of lead SNPs for each QTL. The method is demonstrated by application to three milk yield traits in Nordic Holstein cattle. Many previously identified loci were also confirmed here. Furthermore, we used the mammalian phenotype database to help to find the candidate genes and Variant Effect Predictor (VEP) annotations to screen for possible causal mutations.

## Results

We applied a GWAS analysis approach that automatically and iteratively accounts for the effects of QTL identified in previous iteration(s), a similar approach to conditional analysis implemented in GCTA [11]. The impact of pre-correction on type I error rate was assessed by analyzing simulated data with the effect of a quantitative trait nucleotide (QTN) added to the real phenotypic data (for details on simulation, see Method section). The candidate genes were picked as the closest genes to the lead SNP and listed in Tables 1, 2 and 3. The search for candidate genes started with the top SNP location. However, the whole genomic region showing strong associations with the trait was searched, as the top SNP may not be always located closest to the causal gene due to differences in: LD, imputation accuracy and minor allele frequency. Therefore, we included discussion on other relevant genes (based on association results, known gene function etc.) which could be candidate genes underlying the QTL.

**Table 1:** Lead SNPs from genome-wide associated regions for fat yield in Nordic Holstein cattle. Base positions are given as position in UMD 3.1.1 [49]

BTA	base position	Imputation accuracy	Effect	–log_10_(p)	Region	Gene	Annotation
1	71,227,484	0.9745	−1.77	9.66	70,442,929~71,477,578	*TNK2*	intron
2	126,979,882	0.9972	−1.31	11.46	126,041,707~127,230,335	*PIGV* (near)	downstream
2	85991577^b^	0.9542	1.30	8.91	85,042,155~86,241,732	*ANKRD44*	intron
3	7,226,390	0.9998	−1.09	9.01	6,264,604~7,476,497	*NOS1AP*	intron
5	93,948,357	0.9906	3.28	62.41	93,698,481~94,198,670	*MGST1*	intron
5	20284735^b^	0.9692	−1.30	9.79	20,035,379~20,534,779	5S_rRNA (near)	intergenic
6	95,497,933	0.9996	−1.45	14.76	95,248,213~95,747,954	*PAQR3* (near)	intergenic
6	32950721^b^	0.4975	6.33	11.39	32,367,171~33,200,834	*ENSBTAG00000047255*	intron
7	57,287,990	0.8807	−1.66	20.11	57,038,215~57,538,309	*KCTD16*	intron
9	38,715,137	0.9809	−1.47	8.89	38,345,408~38,965,425	*LAMA4*	intron
11	88,771,449	0.9876	1.16	10.43	88,521,462~89,021,477	*ENSBTAG00000047976* (near)	intergenic
11	15323223^b^	0.8962	−1.32	9.81	14,855,568~15,573,444	*TTC27*	intron
12	68,965,758	0.9957	−1.10	8.93	68,502,223~69,216,445	*ENSBTAG00000045195* (near)	intergenic
14^a^	1,802,265	0.9398	−6.93	240.56	1,549,133~2,049,435	*DGAT1*	missense
14^a^	1,802,266	0.9362	−6.93	240.56	1,549,133~2,049,435	*DGAT1*	missense
14	67981742^b^	0.7652	1.65	8.71	67,117,232~68,231,920	*STK3*	intron
14	1321721^c^	0.4442	1.46	8.82	1,087,168~1,583,427	*ENSBTAG00000046435*	missense
15	65,891,100	0.9992	1.50	12.99	65,641,131~66,141,839	*ELF5* (near)	intergenic
15	25044706^b^	0.9908	−1.17	9.80	24,795,472~25,295,470	*ZBTB16*	intron
16	31,496,700	0.9501	−1.37	9.32	30,519,873~31,746,789	*CNST*	intron
17	62,543,160	0.9898	1.14	10.49	62,224,291~62,793,298	*TBX5*	intron
18	18,970,551	0.9442	−1.19	10.30	18,341,203~19,220,732	*NKD1* (near)	intergenic
19	27,522,927	0.8500	−1.32	10.86	26,625,240~27,773,922	*ASGR1* (near)	intergenic
20	22,609,736	0.9813	1.53	14.23	21,664,412~22,859,809	*MAP3K1* (near)	intergenic
20	44186112^b^	0.9997	1.53	10.20	43,936,468~44,436,133	*ENSBTAG00000040572* (near)	intergenic
26	20,547,445	0.9993	−1.76	21.46	20,299,309~20,797,570	*COX15*	intron
26	42408595^b^	0.9998	−1.21	10.30	41,409,014~42,658,925	*TACC2*	intron
29	23,609,412	0.7717	2.06	10.73	22,613,737~23,859,451	*ENSBTAG00000047094* (near)	intergenic
Total number of significant SNPs					52,334		

^a^Fourteen additional SNPs on chromosome 14 located near *DGAT1* gene had same highest P value (details on those not presented). Note, ^b^indicated this SNP was found on second round, ^c^indicated this SNP was found on third round

**Table 2:** Lead SNPs from genome-wide associated regions for protein yield in Nordic Holstein cattle. Base positions are given as position in UMD 3.1.1 [49]

BTA	base position	Imputation accuracy	Effect	–log_10_(p)	Region	gene	Annotation
1	63,177,947	0.9885	−1.94	12.35	62,590,679~63,428,175	*ENSBTAG00000046854* (near)	intergenic
2	124,837,669	0.9886	1.59	12.63	124,587,873~125,089,732	*PTPRU*	intron
2	86095020^a^	0.9910	1.27	9.53	85,393,563~86,345,056	*ANKRD44*	intron
3	17,160,521	0.9717	−1.15	8.76	16,197,245~17,415,613	*S100A12* (near)	upstream
4	103,211,543	0.9321	−1.06	8.74	102,341,267~103,461,820	*ATP6V0A4*	intron
5	93,511,826	0.8626	−1.37	14.25	93,087,740~93,762,020	*LMO3* (near)	intergenic
5	21792183^a^	0.9813	−1.37	10.39	21,542,557~22,042,238	*SNORD107* (near)	intergenic
5	87923795^b^	0.9926	1.50	8.97	86,950,758~88,173,798	*ETNK1* (near)	intergenic
6	88,477,501	0.9962	−2.60	25.98	88,227,821~88,727,537	*SLC4A4*	intron
6	48,694,003^a^	0.9785	1.89	12.16	47,720,473~48,944,178	ENSBTAG00000045570 (near)	intergenic
6	88847595^b^	0.9009	−1.82	23.84	88,477,501~89,097,608	*GC* (near)	intergenic
7	41,372,989	0.9999	−1.54	18.14	41,085,164~41,623,965	*MGAT1* (near)	intergenic
7	72100619^a^	0.9077	1.59	13.29	71,120,920~72,350,707	*EBF1* (near)	intergenic
8	93,065,787	0.8573	1.65	10.07	92,816,321~93,315,869	*GRIN3A*	Intron
8	31538155^a^	1.0000	1.91	9.62	30,550,864~31,788,181	*LURAP1L* (near)	intergenic
9	33,267,855	0.8655	−1.46	11.96	32,627,954~33,518,971	*SLC35F1* (near)	intergenic
10	93,933,304	0.8370	−1.36	9.90	92,933,459~94,183,400	*SEL1L*	intron
11	35,512,708	0.9999	−1.45	11.82	35,189,581~35,762,749	*ENSBTAG00000027786* (near)	intergenic
13	37,208,792	0.9279	−1.69	10.90	36,702,834~37,459,042	*MKX* (near)	intergenic
14	1,835,440	0.7471	2.84	48.66	1,448,510~2,085,468	*BOP1*	intron
14	67981742^a^	0.7652	1.78	11.60	67,731,848~68,231,920	*STK3*	intron
16	32,262,983	0.9290	−1.52	12.79	31,268,349~32,513,084	*SMYD3*	intron
18	57,015,407	0.9754	2.56	17.71	56,767,474~57,265,703	*POLD1*	intron
18	15057077^a^	0.9934	1.27	9.99	14,811,219~15,308,407	*VPS35*	intron
19	27,522,927	0.8500	−1.42	12.55	27,156,952~27,773,922	*ASGR1* (near)	intergenic
19	61014793^a^	0.8505	−1.08	8.65	60,313,953~61,265,218	*KCNJ2* (near)	intergenic
20	69,006,609	0.9920	−1.29	11.27	68,120,719~69,256,618	*IRX1* (near)	intergenic
20	8830351^a^	0.9433	−1.71	10.61	8,345,063~9,080,402	*ENSBTAG00000012775* (near)	intergenic
23	10,974,968	0.9304	−1.18	10.68	10,234,192~11,224,969	*FGD2* (near)	intergenic
25	36,403,719	1.0000	1.33	10.25	36,112,575~36,654,175	*EPO* (near)	intergenic
26	37,695,494	0.9122	−1.41	14.76	36,699,144~37,945,656	*SHTN1* (near)	intergenic
27	36,304,978	0.9834	1.06	8.52	36,037,123~36,555,106	*ANK1*	intron
29	17,620,617	0.9576	1.47	10.37	16,671,270~17,870,637	*NARS2*	intron
29	35459126^a^	0.9999	1.61	10.11	34,854,011~35,709,168	*NTM*	intron
Total number of significant SNPs					36,644		

Note, ^a^indicated this SNP was found on second round, ^b^indicated this SNP was found on third round

**Table 3:** Lead SNP from genome-wide associated regions for milk yield in Nordic Holstein cattle. Base positions are given as position in UMD 3.1.1 [49]

BTA	base position	Imputation accuracy	Effect	–log_10_(p)	Region	Gene	Annotation
2	80,753,895	0.9454	1.13	9.95	79,777,813~81,003,948	*NABP1* (near)	intergenic
3	56,402,959	0.9308	−1.36	11.68	56,152,966~56,653,364	*ENSBTAG00000001873* (near)	intergenic
4	101,547,644	0.7008	−1.66	12.65	100,921,921~101,798,041	*CHRM2* (near)	upstream
5	93,953,487	0.9726	−2.10	29.52	93,703,737~94,203,599	*MGST1* (near)	upstream
5	31005518^b^	0.9943	1.42	12.25	30,202,453~31,258,920	*WNT1* (near)	upstream
5	85080296^c^	0.7619	−1.28	11.24	84,425,435~85,330,671	*KRAS* (near)	intergenic
5	20569435^d^	0.9944	1.23	9.37	19,600,731~20,820,066	*CCER1* (near)	intergenic
6	88,847,595	0.9009	−1.78	21.61	88,598,011~89,097,608	*GC* (near)	intergenic
6	46901490^b^	0.7413	−1.28	11.45	46,181,675~47,152,919	*SEL1L3* (near)	intergenic
6	38027010^c^	0.9950	−4.75	9.47	37,669,181~38,279,802	*ABCG2*	missense
7	65,370,850	0.9848	−1.36	13.58	65,120,872~65,620,985	*GLRA1* (near)	intergenic
8	73,877,814	0.8453	−1.37	11.14	73,629,406~74,127,901	*ENSBTAG00000010829* (near)	upstream
8	42062591^b^	0.9595	−1.27	10.07	41,064,643~42,313,291	*KCNV2* (near)	intergenic
9	33,478,527	0.8801	−1.25	9.23	32,627,954~33,728,755	*ENSBTAG00000015497* (near)	intergenic
10	1,989,907	0.9469	−1.15	9.92	1,016,031~2,240,288	*ENSBTAG00000047622* (near)	intergenic
13	36,822,330	0.9933	−1.66	10.74	36,572,364~37,072,486	*MPP7*	intron
14^a^	1,802,667	0.7975	5.98	178.35	1,545,264~2,044,412	*DGAT1*	intron
15	54,392,611	0.9577	1.57	16.58	53,485,007~54,642,856	*PPME1*	intron
16	28,384,260	0.9984	1.64	10.50	28,012,864~28,634,313	*CNIH3* (near)	intergenic
17	66,510,224	0.9438	1.83	11.63	66,119,023~66,760,263	*CORO1C*	intron
18	46,583,346	0.9829	1.86	11.97	46,333,384~46,833,392	*UPK1A* (near)	upstream
19	27,442,452	0.7904	−1.26	9.71	26,592,355~27,692,965	bta-mir-497 (near)	downstream
20	29,996,719	0.9580	−2.95	31.02	29,748,423~30,246,822	*MRPS30* (near)	intergenic
23	25,076,472	0.9797	−1.34	9.23	24,219,868~25,326,583	*GCM1*	intron
26	37,716,420	0.9790	−1.43	12.28	36,730,021~ 37,966,463	*SHTN1* (near)	intergenic
28	34,972,377	0.9991	1.18	9.81	34,722,402~35,222,855	*ZMIZ1* (near)	intergenic
Total number of significant SNPs					55,600		

^a^Eight additional SNPs on chromosome 14 had same highest *P* value. Note, ^b^indicated this SNP was found on second round, ^c^indicated this SNP was found on third round, ^d^indicated this SNP was found on fourth round

Our approach of including associated SNPs as covariates in subsequent rounds of analyses did not increase the type I error rates. We simulated one SNP as a QTN and considered 10 other SNPs with different levels of LD (r^2^) with the QTN in order to test whether our method introduces type I error into analysis when fixing lead SNPs detected in previous iterations as covariates [12]. We generated new phenotypes from the real phenotypic value plus the simulated QTN effects. The QTN’s contribution to individuals’ phenotypes was obtained by multiplying the genotype dosage of the QTN with the allele substitution effect which was drawn from a normal distribution with a mean 20% of the standard deviation (SD) of the phenotype and variance as 1% of the phenotypic variance. The simulation was repelicated 100 times. We detected the simulated QTN as the lead SNP in the first round of all 100 replicates. When the simulated QTN was included in the model as a covariate, we did not observed any of the 10 SNPs in LD with QTN to be significant (i.e., no false positives detected).

### The GWAS of fat yield

**Fig. 1:**
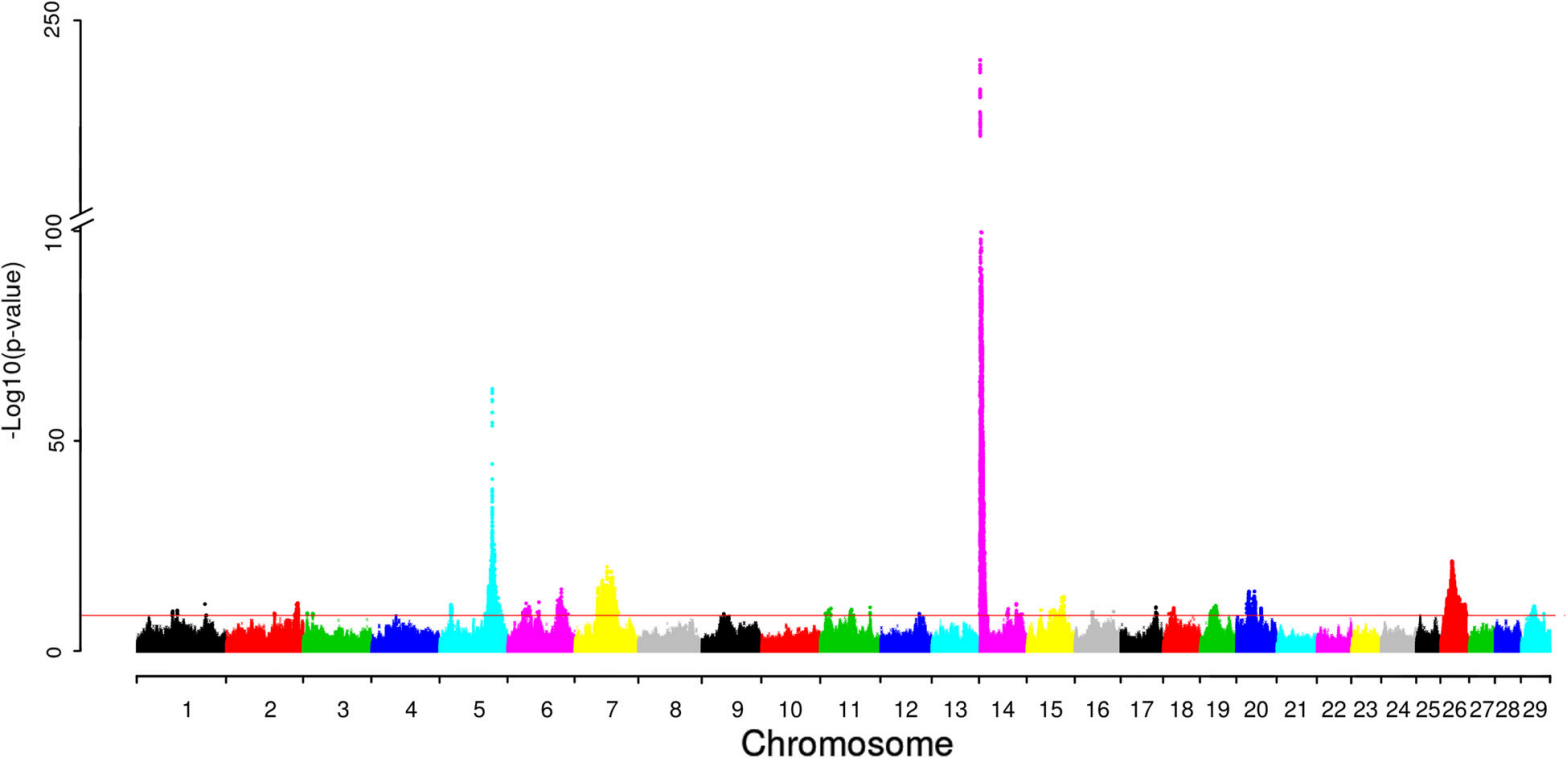
Manhattan plot for association of SNP with fat yield in Nordic Holstein cattle. Red horizontal line indicates genome-wide significance level [−log10(P) = 8.5]

Analyzing milk fat yield, our approach detected nine additional QTL over and above the QTL detected in the first round (Fig. 1 and Table 1). In Table 1, the first SNP on each chromosome is the lead SNP from the first round of GWAS analysis, the rest are the additional SNP(s) detected on a chromosome. Sixteen SNPs on chromosome 14 have the same *P*-value in the first round, and these SNPs are in high LD with the two known causative polymorphisms in *DGAT1* [13], BTA14: 1802265 (rs109234250) and BTA14: 1802266 (rs109326954) (Additional file 1: Figure S1). The variant effect predictor (VEP) [14] annotation showed these two variants in *DGAT1* are missense mutations. The second strongest association signal was located on chromosome 5 with lead SNP, BTA5: 93948357 (rs209372883) located within the intron of *MGST1*. *MGST1* was previously reported associated with the milk fat content [15]. On chromosome 26, our lead SNP pointed to *COX15.* In a human study, this gene was proposed involved in biosynthesis of heme A [16]. Even though this gene is a promising positional candidate gene, no biological information currently links this gene to milk fat yield. Another gene known to affect milk fat content is *SCD1* [17] located at chromosome 26: 21141592 ~ 21,148,318. Our lead SNP on chromosome 26 (BTA26:20547445, rs136702635) is located close to it. We estimated the variance explained by QTL. The QTL (18 QTL) found from the first round explained 23.56% of the variance of de-regressed proof breeding value (DRP) for fat yield and all QTL (27 QTL) explained 28.57% of the DRP variance (Table 4).

**Table 4:** The genetics variants explained by QTL and the rest of SNPs

	Number of QTL	V(G1)/Vp^b^ (%)	V(G2)/Vp^c^ (%)
Fat1^a^	18	23.56	61.12
Fat2^a^	27	28.57	56.40
Prot1^a^	22	12.52	72.20
Prot2^a^	34	16.76	67.14
Milk1^a^	20	19.02	66.27
Milk2^a^	26	21.50	63.12

Note, ^a^Fat means the trait of fat yield, Prot means the trait of protein yield, Milk means the trait of milk yield; 1 indicate the lead SNP list only included the lead SNP from the first round, 2 indicated the lead SNP list included all lead SNP found by our approach. ^b^means the percentage of genetics variants explained by the QTL, ^c^ means the percentage of genetics variants explained by the rest of SNP other than QTL

### The GWAS for protein yield

**Fig. 2:**
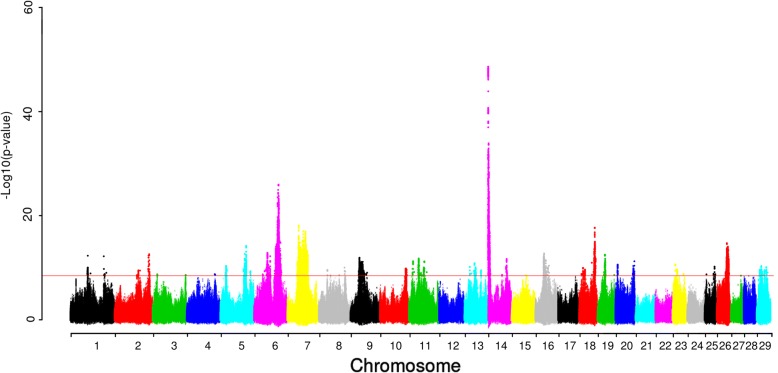
Manhattan plot for association of SNP with protein yield in Nordic Holstein cattle. Red horizontal line indicates genome-wide significance level [−log10(P) = 8.5]

We ran the analysis on the milk protein yield (Fig. 2), and found 34 lead SNPs (Table 2), 12 of which were detected in the second or third round. The strongest association signal for protein yield was on BTA14 with lead SNP BTA14:1835440 (rs208567981), located within *BOP1*. The annotation of BTA14:1835440 (rs208567981) is a missense mutation, and the SIFT annotation is tolerant. However, this signal is most likely due to the known mutation in *DGAT1*. The lead SNP (rs208567981) was in strong LD with SNPs located within *DGAT1* and the –log_10_(P) value of these 19 SNPs within *DGAT1* were larger than 47.99 (including two known causative variants in *DGAT1*, Additional file 1: Figure S2). This result shows that the causal mutation may not necessarily be the SNP in highest association. The second lead SNP of this analysis is BTA6: 88477501, which is located near the well-studied casein genes *CSN1S1*, *CSN1S2*, *CSN3* and *CSN2* [18]. We estimated the variance explained by QTL. The QTL (22 QTL) found only from the first round explained 12.52% of the DRP variance for protein yield and all QTL (34 QTL) explained 16.76% of the DRP variance (Table 4).

### The GWAS for milk yield

**Fig. 3:**
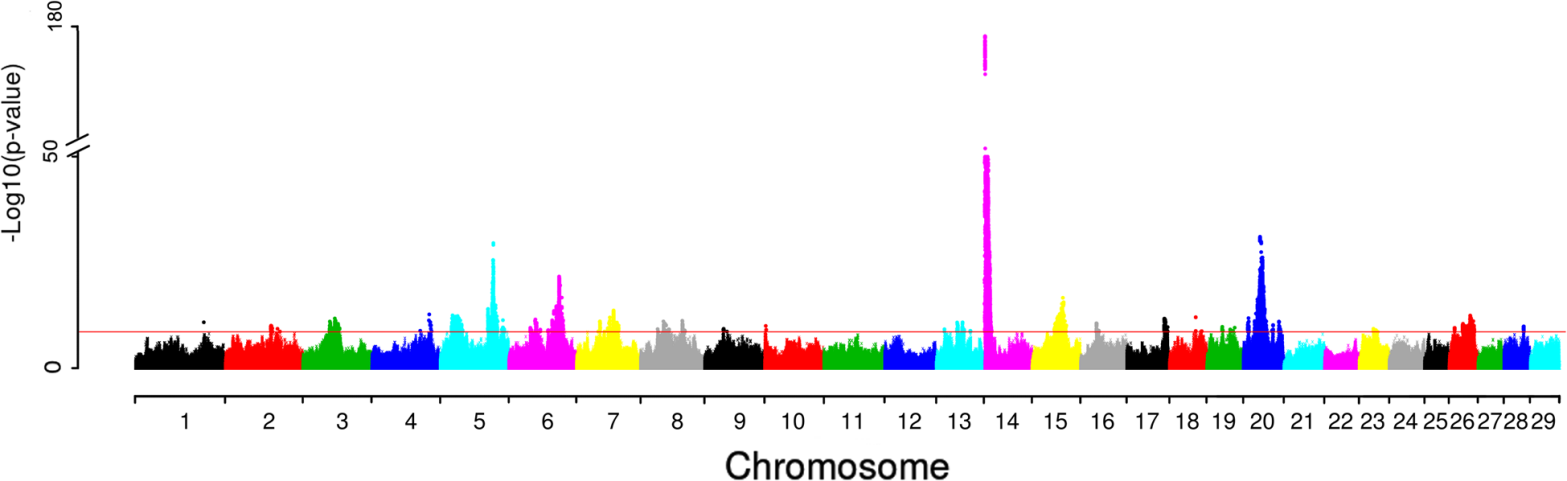
Manhattan plot for association of SNP with milk yield in Nordic Holstein cattle. Red horizontal line indicates genome-wide significance level [−log10(P) = 8.5]

We applied our analysis to milk yield (Fig. 3). A total of 26 lead SNPs (Table 3) were detected, out of which six were detected in the second, third or fourth round. The most significant association signal was in the *DGAT1* gene. The second most significant association signal was at BTA20:29996719 (rs43116343), which is close to *MRPS30*. A recent study showed *MRPS30* to be associated with lactation persistence in Canadian Holstein cattle [19]. This lead SNP is also close to the growth hormone receptor, *GHR* [20]. The causative mutation of *GHR* is BTA20:31909478 (rs385640152), and is known to affect milk yield [20]. The third strongest lead SNP was BTA5:93953487 (rs210234664). This SNP is close to *MGST1*. A previous eQTL study showed *MGST1* may affect milk composition [21]. With our approach, we detected BTA6: 38027010 (rs43702337) in the third round, located in *ABCG2*. *ABCG2* was previously reported to affect milk yield in dairy cattle [22]. This SNP is a missense variant; its SIFT annotation is “deleterious” and has previously been proposed as a causative mutation [23]. We estimated the variance explained by QTL. The QTL (20 QTL) found from the first round explained 19.02% of the DRP variance for milk yield and all QTL (26 QTL) explained 21.50% of the phenotypic variance (Table 4).

### Post-GWAS analysis using the mammalian phenotype database

The criteria for selecting positional candidate genes was the gene located closest to the lead SNP. For future identification and research on genes biologically associated with milk traits, we tried to find whether there are other genes which should be considered as potential candidate genes other than the candidate gene lists (Tables 1, 2 and 3). Considering the high LD structure of cattle population, the causal genes may be located within the genome region in LD with lead SNPs. One source of additional information that may help to prioritize genes, is to find the link between the gene and the possible function in the mammalian phenotype database related to milk and milk-organ related traits [24]. Therefore, we extracted genes which overlap with the LD region of the lead SNP and search them in the mammalian phenotype database [24]. We only paid attention to two kinds of phenotypes: “abnormal mammary gland development” or “abnormal milk composition”. Ten genes from the GWAS hits were also annotated as related to these two types of phenotype. This annotation appears to have biological relevance, although the enrichment of these 10 genes in the mammalian phenotype database analyzed by Fishers’ exact test was not significant. The results showed five genes were reported to be related with “abnormal milk composition” (Table 5). Out of this list, *CSN1S1*, *CSN2*, *CSN3* and *DGAT1* were reported in dairy cattle and also identified in the present study. Furthermore, we identified six genes related to “abnormal in mammary gland development” (Table 6) in mammalian phenotype database. In this list *DGAT1* showed abnormal phenotype in both kinds of phenotype description we searched. In addition to the well-studied genes (*CSN1S1*, *CSN2*, *CSN3* and *DGAT1*), the remaining five genes are *ELF5*, *CAT, STK3*, *CHUK,* and *WNT1. ELF5* is one of the candidate genes proposed by the closest genes to lead SNP (BTA15: 65891100) associated with the fat yield (Table 1). *ELF5* was previously found related to mouse mammary development [25] and may also influence the milk content through milk protein synthesis in cattle [26]. *CAT* is also located close to the same lead SNP as *ELF5*. *CAT* is involved in several biological processes including GO term ‘responds to fatty acid’ [27]. *CHUK,* close to BTA26: 20547445, is associated with fat yield (Table 1). This gene is known as a key gene involved in mammary development in mice [28]. *STK3* is the nearest gene to the second lead SNP (BTA14: 67981742) on the same chromosome associated with milk protein yield (Table 2). This gene was found to play a pivot role in controlling cell proliferation [29] and tumor suppression [30] in human studies. *WNT1* is the nearest gene to the second lead SNP of milk yield (Table 3).

**Table 5:** Genes related to “abnormal milk composition” phenotype in the mammalian phenotype database [24] overlapped with milk QTL identified in the present study

Gene name	Location	Phenotype
*CSN1S1*	BTA6: 87,141,556-87,159,096	abnormal milk composition
*CSN2*	BTA6: 87,179,502-87,188,025	abnormal milk composition
*CSN3*	BTA6: 87,378,398-87,392,750	abnormal milk composition
*DGAT1*	BTA14: 1,795,351-1,804,562	abnormal milk composition
*IL4I1*	BTA18: 56,691,667-56,725,849	abnormal milk composition

**Table 6:** Genes related to “abnormal of mammary gland development” in the mammalian phenotype database [24] overlapped with milk QTL identified in the present study

Gene name	Location	Phenotype
*WNT1*	BTA5: 31,000,183- 31,003,266	abnormal mammary gland morphology
*CAT*	BTA15: 65,779,325-65,815,261	decreased mammary gland tumor incidence
*ELF5*	BTA15: 65,824,442-65,854,386	abnormal mammary gland development
*STK3*	BTA14: 67,677,676-67,987,801	increased mammary gland tumor incidence
*DGAT1*	BTA14: 1,795,351-1,804,562	abnormal mammary gland development
*CHUK*	BTA26: 20,966,010-21,008,277	abnormal mammary gland growth during pregnancy

### Annotation of SNPs in LD with lead SNPs

**Fig. 4:**
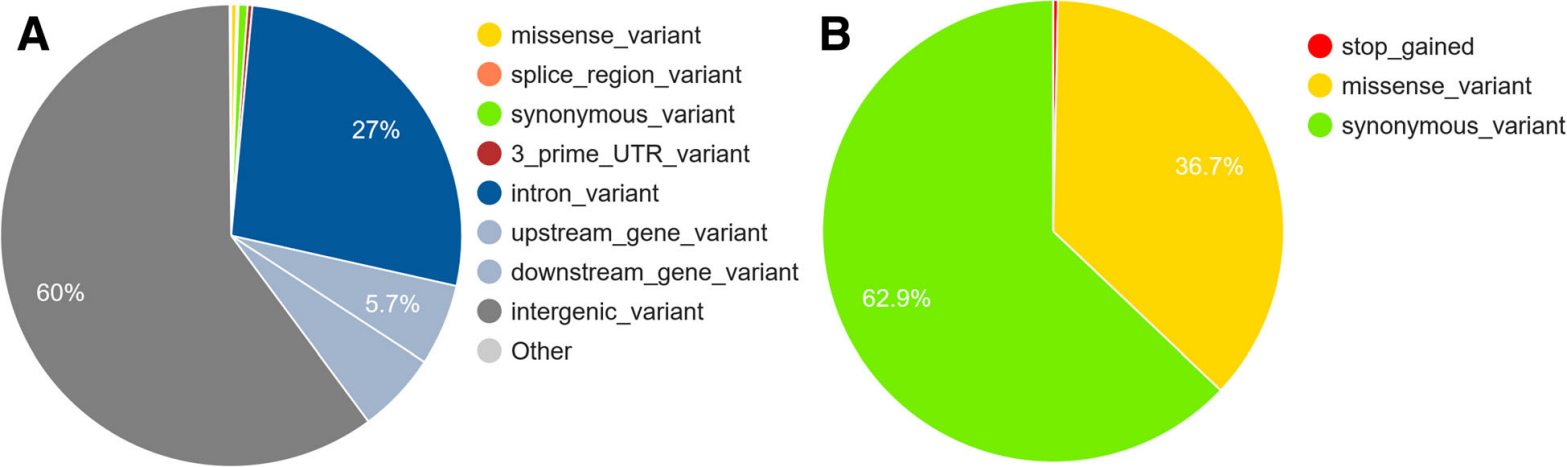
The VEP annotation of SNPs in linkage disequilibrium (LD > 0.20) with leading SNPs. **a** The summary of all annotation. **b** The summary of annotation that change the protein coding sequence

As shown before, the causative mutation maybe located in the neighboring region of the lead SNP. Therefore, we extracted all SNPs in LD with leading SNPs (r^2^ > 0.2) and annotated them using VEP [14]. We extracted 27,612 SNPs and obtained 29,249 annotations (because some genes or transcripts overlap). The majority of these SNPs are intergenic variants or intron variants (Fig. 4a). Among the SNPs that changed the coding sequence of the protein, most of them were synonymous variants (Fig. 4b). Using this result, we checked if we could prioritize candidate mutations in the candidate genes. For example *GHR*, the well-known causative mutation for *GHR* is BTA20:31909478 (rs385640152, F279Y) [20]. The annotation for this SNP is a missense mutation and the SIFT score is 0.02 which is ‘deleterious’.

Further, we checked whether we can detect some candidate mutations in the new candidate genes. Four genes (*CSN1S1*, *CSN2*, *CSN3* and *DGAT1*) were found related to abnormal milk composition and *DGAT1* related to mammary gland development (Table 5 and Table 6) as reported previously. In addition to *DGAT1*, we found one tolerance missense mutations in *CSN2*. Moreover, in *IL4I1*, we found one deleterious missense mutations.

## Discussion

Although functional gene clustering is weaker in eukaryotes genomes than in prokaryotes genomes, functional grouping of the genes with same or similar function still exists [31]. Therefore, in GWAS analysis, we may fail to detect the nearby genes and may treat them as one significant signal. In our study, we used an analysis approach to detect multiple nearby QTL by iteratively fixing the lead SNP as covariate. However, such an approach can inflate type I error rate [12]. To avoid introducing additional type I errors, we placed a condition that the lead SNPs detected in the second and subsequent rounds must be found to be genome-wide significant in the first round (i.e., significant according to conventional GWAS criteria). In addition, we tested our approach on simulated data with a simulated QTN and multiple SNPs with various levels of LD with the QTN. In 100 replicates, we found no additional SNP in LD with the QTN other than the simulated causative variants. By using this analysis, we were able to detect multiple QTL (as well as designating the lead SNP for each QTL) on a chromosome automatically. For example, we detected a known QTL on BTA6 (BTA6:38027010, rs43702337) in the third round and also another QTL at 46 Mb (in the second round). This SNP is located in the gene *ABCG2* which was previously reported to affect milk yield in dairy cattle [22] and this lead SNP was the most probable causative mutation [23]. Furthermore, our approach also showed the potential to distinguish closely linked QTL. For example the lead SNPs on chromosome 6 of protein content, we detected the first association signal at BTA6: 88477501 and the third association signal at BTA6: 88749792. Similar conditional analyses were also applied in human and other livestock studies [32–34]. Here, we analyzed one lead SNP at a time, as opposed to Bolormaa et al. [34] who included all lead SNPs simultaneously in the model. We also compared the genetic variants explained by the QTL found by first round and all the QTL found by our approach. The results showed the QTL found at second and third round did explain more phenotype variants (Table 4).

Post GWAS, we face the challenge of identifying the candidate genes. The conventional method is to use the nearest gene, but this may miss the target as many-a-time the lead SNP may not be from the causal gene. This could be due to imputation inaccuracies, multiple QTL in the vicinity or random chance factor. Therefore, we need to use additional information to prioritize the candidate genes. In this study, we used the mammalian phenotype database to search for candidate genes from the genes located in association regions. The mammalian phenotype is based on mouse mutation lines. As a test, we extracted all genes located within LD of the lead SNPs for all three milk yield traits and searched for related phenotype terms. Here, we searched for two phenotype terms ‘abnormal mammary gland development’ and ‘abnormal milk composition’. We successfully identified some well-known genes affecting milk related traits in cattle as well as new candidate genes (Table 5 and Table 6). For the term ‘abnormal milk composition’, we identified five genes. Four of them were reported previously in different studies [35, 36], and only *DGAT1* is the nearest gene to the lead SNP on chromosome 14. Another term we searched is ‘abnormal in mammary gland development’ and found six genes. *ELF5*, *STK3* and *WNT1* are the nearest genes to the lead SNPs. However, differences between mice and cattle may introduce some false positives. In all, using this strategy we not only found some well-studied genes missing from the nearest genes method (pick the gene which is nearest to lead SNP as candidate genes), but also identified new candidate genes which may be helpful in finding causal factors.

We also face another challenge of identifying the causative variant once the causal gene is identified as levels of linkage disequilibrium in cattle are high [37]. In many cases the causative variant is not the lead SNP [38] but another SNP hidden within the LD of the lead SNP. In human studies, there are different strategies to prioritize variants [10]. In this study, information from Ensembl [14] was used to prioritize potential causative variants. In our case, the *DGAT1* and *ABCG2* can be detected in our lead SNP list, and the causative mutation of both can be detected in VEP annotation as missense variants. *GHR* was found nearby the location of lead SNPs. For *ABCG2* and *GHR*, the SIFT score show these mutations as ‘deleterious’. For *DGAT1*, even though the SIFT showed these two mutations are tolerated the amino acid of the protein is changed. Therefore, the impact of moderate and high reported by VEP can be considered as possible causative mutations, while SIFT score can be used to provide additional support.

In summary, our analysis approach can distinguish nearby association signals of multiple QTL. In our study, we found candidate genes reported by previous studies. Followed by searching genes within the LD region of the lead SNPs, we can find high confidence candidate genes. Lastly, using VEP can help us to find putative causative mutations within candidate genes and provides a good source for further functional validation. However, our approach will not be able to pinpoint causal variants located in the non-coding and regulatory regions due to lack of annotation of the cattle genome.

## Conclusion

In this study, we designed an approach for detecting closely linked multiple association signals and performed the analysis in Nordic Holstein cattle for milk, fat and protein yields. The results showed we not only detected most of the well-known genes affecting these three milk yield traits but also detected additional candidate genes. Post-GWAS, we used information from the mammalian phenotype database and variant effect predictor to confirm known genes and causative mutations. In the meanwhile, we detected additional genes which might be contributing to variation in milk traits in Nordic Holstein cattle. Therefore, we concluded our approach can be used routinely for GWAS studies in dairy cattle.

## Methods

### Phenotype and genotype data

No animal experiments were performed in this study, and therefore, approval from the ethics committee was not required.

Phenotypic records for Nordic Holstein cattle are kept in a centralized database (Nordic Cattle Genetic Evaluation, NAV. http://www.nordicebv.info/). Breeding values for milk, fat and protein yield (MY, FY and PY) are based on production figures expressed in kilograms taken from routine milk records and then combined into an index for each trait. For details on genetic evaluation for milk yield traits in Nordic countries see (http://www.nordicebv.info/production). The breeding values used for association analysis were de-regressed proof breeding values [39, 40] from the routine genetic evaluation by NAV and were available for 5043 progeny tested Holstein bulls.

The association study was carried out by using imputed WGS data, as previously described by Iso-Touru et al. [41] and Wu et al. [42]. A total of 4921 bulls were genotyped with the Illumina BovineSNP50 BeadChip (54 k) ver. 1 or 2 (Illumina, San Diego, CA, USA). The 54 k genotypes were imputed to WGS variants by a 2-step approach [43]. First, all animals were imputed to the high-density (HD) level by using a multibreed reference of 3383 animals (1222 Holsteins, 1326 Nordic Red Dairy Cattle, and 835 Danish Jerseys), which had been genotyped with the Illumina BovineHD BeadChip. Subsequently, these imputed HD genotypes were imputed to the WGS level by using a multibreed reference of 1228 animals from *Run4* of the 1000 Bull Genomes Project [1] (1148 cattle, including 288 individuals from the global Holstein population, 56 Nordic Red Dairy Cattle, 61 Jerseys, and 743 cattle from other breeds [1] and additional data from Aarhus University (80 individuals, including 23 Holsteins, 30 Nordic Red Dairy Cattle, and 27 Danish Jerseys).

Different variant calling pipelines were used for the 1000 Bull Genome Project data and the in-house Nordic data at Aarhus University. The whole genome sequence data at Aarhus University was analyzed as described by Brøndum et al. [44]; while the same for 1000 Bull Genome Project was described by Daetwyler et al. [1]. Detailed guidelines are available at http://www.1000bullgenomes.com. Data from both sources were available as VCF files. The data from the two sources were combined using Picards MergeVCFs (https://broadinstitute.github.io/picard/). As the 1000 Bull Genomes Project only shares data after variant calling, some markers were not called for all animals in the combined dataset. To avoid large gaps of missing markers in the dataset, only markers that were called in both the Nordic and the 1000 Bull Genomes Project datasets were kept. For positions containing both a SNP and an INDEL, the INDEL was discarded, as the imputation methods rely on unambiguous sequences of variants. Positions with disagreements between alleles for sequence and HD data were also deleted. Reference genotype probability data was run through BEAGLE [45] and all markers with an R^2^ value (squared correlation between the true and imputed allele dosages) below 0.9 were removed from the original sequence data. This was done in order to remove poorly imputed markers that might have adverse effects on the imputation procedures.

Imputation from 54 k to HD genotypes to HD and imputation to the WGS level were undertaken with IMPUTE2 v2.3.1 [46] and Minimac2 [47], respectively. The imputation to whole genome sequence was done in chunks of 5 Mb with the length of buffer region of 0.25 Mb on either side. A total of 22,751,039 biallelic variants were present in the imputed sequence data. After excluding SNP with a minor allele frequency below 1% or with large deviation from Hardy–Weinberg proportions (*P* < 1.0^− 6^), 15,512,960 SNPs for fat yield, 15,551,720 SNPs for protein yield and 15,551,614 SNPs for milk yield on 29 autosomes in Nordic Holstein cattle were retained for association analyses. The average accuracy (r^2^-values from Minimac2) was 0.85 for across breed imputation. Information on the distribution of imputation accuracy as a function of minor allele frequency has previously been published [42].

### The methodology of multiple QTL detection

We developed an analysis approach to run the conditional GWAS analysis, similar to the *GCTA-COJO* approach in GCTA [11]. However, GCTA-COJO uses GWAS summary data while we have reanalyzed the data after fitting only the lead SNP(s) on a chromosome. Furthermore, we used imputed dosage data instead of number of copies of the reference allele. This takes account of inaccuracies in genotype imputation. We first performed a single SNP GWAS analysis using GCTA [11] for each chromosome as the first round. Then we ranked the SNP based on their –log_10_*P* value in the GWAS. The SNP with the largest –log_10_P value, the lead SNP, within each chromosome was identified. An experiment-wise 0.05 type I error rate after Bonferroni correction for 15,512,960~15,551,720 simultaneous tests corresponds to a threshold of –log_10_P ≈ 8.5. If the –log_10_P value of the lead SNP exceeded 8.5; we extracted the lead SNP’s genotype dosage, fitted it as a covariate, and scanned the whole chromosome again as the second round. If the result of second round detected another SNP with a –log_10_P value exceeding 8.5 and this SNP also was significant in the first round (–log_10_P > 8.5), we extracted the allele dosage of this SNP and fixed it as another covariate and scanned the chromosome in a third round. This same procedure was iterated until no additional SNP remained significant. The lead SNP in each round were collected to build a lead SNP list. Moreover, in each round solo SNP, that is, SNP with no other significant SNP within a 1 Mb region were removed. A boundary for each QTL peak was defined as follows: for each QTL, we scanned the 1 Mb region up- and down-stream of each lead SNP, if SNP –log_10_P value decreased by more than 3 units compared to the value at the leading SNP and the region is larger than 0.25 Mb we set this SNP as a boundary, otherwise we set ±0.25 Mb as the boundary. The list of candidate genes were generated from the closest annotated genome feature to the lead SNP list.

### Testing the type I error rate using simulation data

We used simulated phenotype data to test whether our approach to detect multiple QTL on a chromosome by incorporating previously identified QTL as covariates, inflates the type I error rates [12]. We selected a SNP randomly from the genome as a causative mutation (QTN) with a MAF (Minor Allele Frequency) between 0.05 and 0.10 and in Hardy Weinberg equilibrium. Ten additional SNP with different levels of LD (linkage disequilibrium, r^2^) with the simulated QTN were selected. These 10 SNPs have different r^2^ with the QTN as follows: one with 0.9–1, one with 0.8–0.9, one with 0.8–0.7, one with 0.7–0.6, one with 0.6–0.5, one with 0.5–0.4, one with 0.4–0.3, one with 0.3–0.2 and two with less than 0.2. Allele substitution effects at the QTL were sampled from a univariate normal distribution with mean of 20% of the standard deviation of phenotype and variance equal to 1% of the phenotypic variance. We repeated this simulation and applied our analysis 100 times. Lastly, we investigated how many times we found a SNP in LD with the simulated QTN after we fix the simulated causative mutation as a covariate i.e. false positive detection.

### LD calculation and annotation

We calculated the pairwise r^2^ between lead SNP and all other SNPs on the same chromosome using PLINK [48] and extracted all the SNPs which have r^2^ > 0.2 with the lead SNP. All these SNPs were annotated by VEP (Variant Effect Predictor) [14]. To find the candidate genes, we extracted all the genes which overlap with LD regions of the lead SNP and searched these gene entries in the Mammalian Phenotype database [24]. We collected all the lead SNPs and calculated the pairwise r^2^ with SNPs in the chromosome. The boundary was set to the last SNP that has r^2^ > 0.2. Then we extracted all the genes overlapping these regions and searched them in the database. We found 417 genes located in the LD regions, of which 388 have gene symbols. These 388 genes were searched in the database and 375 have mutation lines with phenotype descriptions in the Mammalian Phenotype database. We refined results using two terms for phenotypes: ‘abnormal in mammary gland development’ and ‘abnormal in milk production’.

### The genetics variants explained by QTL

We used the lead SNP list to generate the genetic relationship matrix (GRM) as group 1. Then we excluded all flank 2.5 Mb SNPs of the lead SNP from the imputed HD data to generate GRM as group 2. At last, we estimated variance explained by these two groups for each trait. The whole analysis was conducted using GCTA [11].

## Additional file

Additional file 1 **Figure S1.** The locuszoom [1] figure of previous report causative mutation of *DGAT1* of the genome-wide association result milk fat yield in Nordic Holstein cattle*.* and **Figure S2.** The locuszoom figure of previous report causative mutation of *DGAT1* of the genome-wide association result in milk protein yield in Nordic Holstein cattle*.BOP1* was not include in USCS refFlat file [2]. (DOCX 551 kb)

## Abbreviations

*ABCG2*: ATP-binding cassette sub-family G member 2
BTA: *Bos taurus* autosome
*CAT*: Catalase
*CHUK*: Inhibitor of nuclear factor kappa-B kinase subunit alpha
*COX15*: Cytochrome c oxidase assembly protein COX15 homolog
*CSN1S1*: Alpha-S1-casein
*CSN1S2*: Alpha-S2-casein
*CSN2*: Beta-casein
*CSN3*: Kappa-casein
*DGAT1*: Diacylglycerol O-acyltransferase 1
*ELF5*: ETS-related transcription factor Elf-5
eQTL: Expression quantitative trait loci
*GHR*: Growth hormone receptor
GWAS: Genome wide association study
LD: Linkage disequilibrium
*MGST1*: Microsomal glutathione S-transferase 1
*MRPS30*: 28S ribosomal protein S30, mitochondrial
NGS: Next-generation sequencing
QTL: Quantitative trait loci
QTN: Quantitative trait nucleotide
*SCD1*: Acyl-CoA desaturase 1
SD: Standard deviation of the phenotype
SIFT: Sorting Intolerant from Tolerant
SNP: Single nucleotide polymorphism
*STK3*: Serine/threonine-protein kinase 3
VEP: Variant Effect Predictor
