# Supplementary material for: Dissecting closely linked association signals in combination with the mammalian phenotype database can identify candidate genes in dairy cattle
Source: BMC Genet. 2019 Jan 29;20:15. doi: 10.1186/s12863-019-0717-0 (PMC6350337; doi:10.1186/s12863-019-0717-0)
Supplement: Supplementary file 1 — Figure S1. The locuszoom [1] figure of previous report causative mutation of DGAT1 of the genome-wide association result milk fat yield in Nordic Holstein cattle. and Figure S2. The locuszoom figure of previous report causative mutation of DGAT1 of the genome-wide association result in milk protein yield in Nordic Holstein cattle.BOP1 was not include in USCS refFlat file [2]. (DOCX 551 kb) [file 12863_2019_717_MOESM1_ESM.docx]

**Distinguishing closely linked association signals and combing mammalian phenotype data can help to identify candidate genes in dairy cattle**

Zexi Cai, Bernt Guldbrandtsen, Mogens Sandø Lund, Goutam Sahana


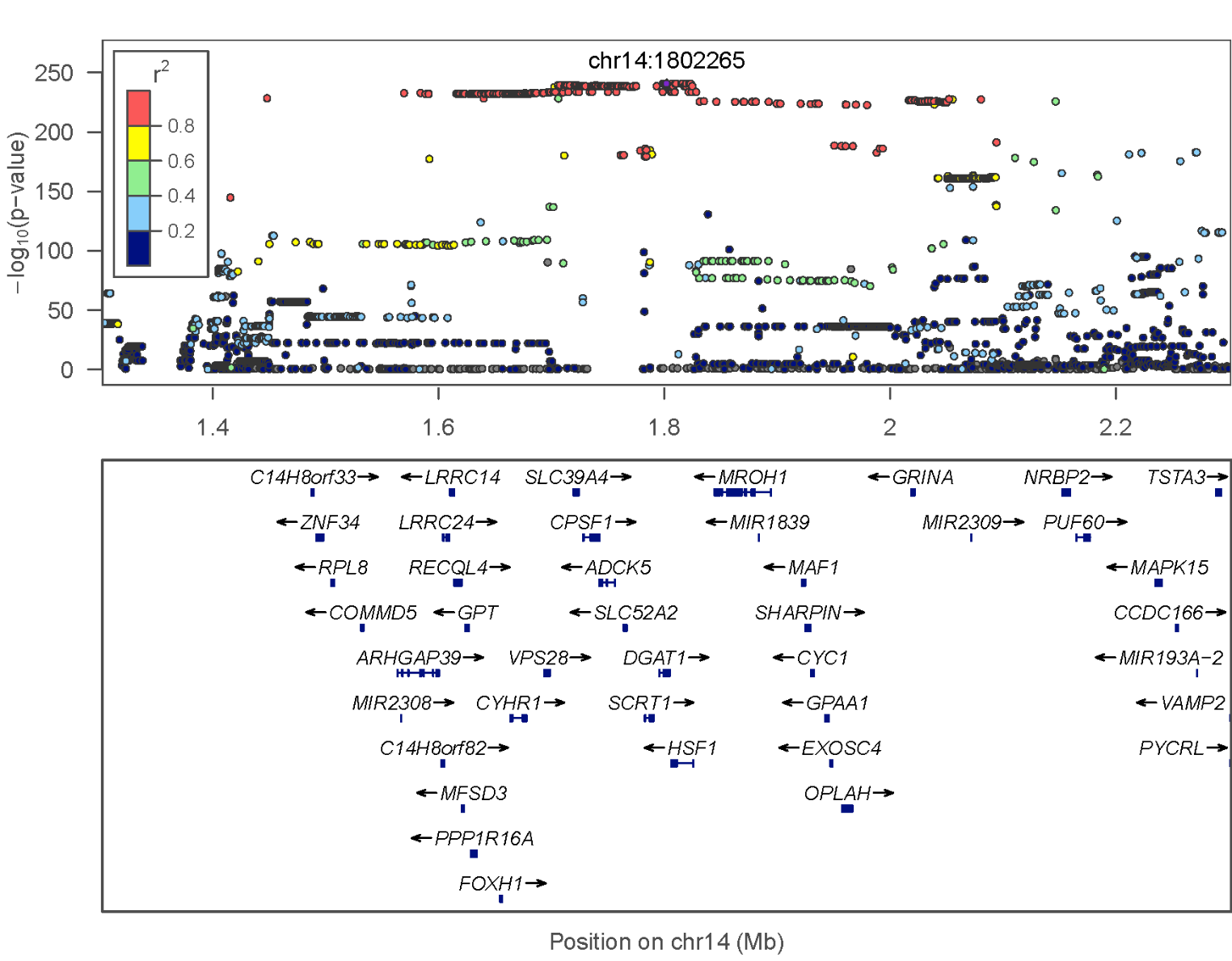


Figure 1S. The locuszoom [1] figure of previous report causative mutation of *DGAT1* of the genome-wide association result milk fat yield in Nordic Holstein cattle*.*


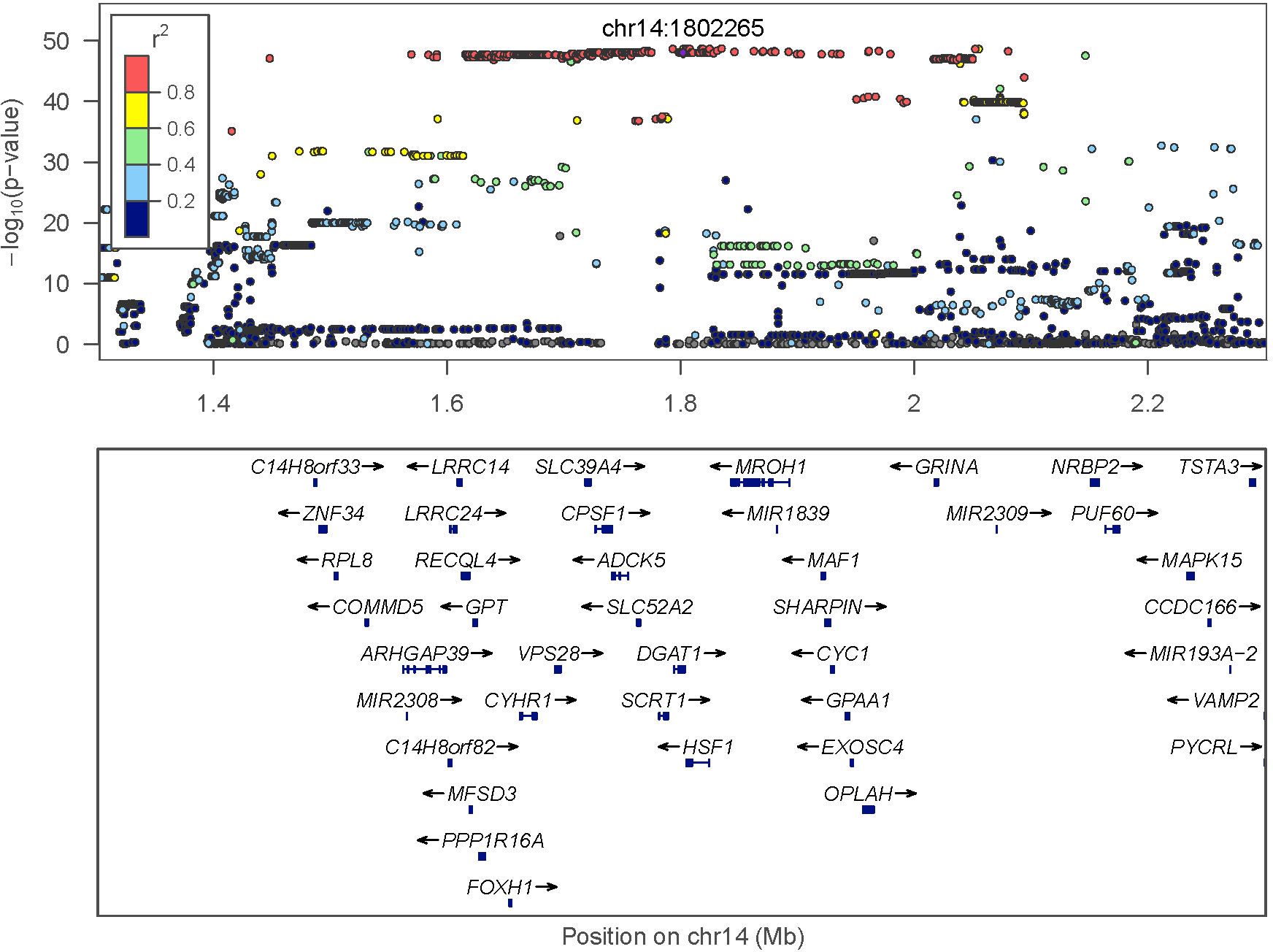


Figure 2S. The locuszoom figure of previous report causative mutation of *DGAT1* of the genome-wide association result in milk protein yield in Nordic Holstein cattle*.BOP1* was not include in USCS refFlat file [2].

1. Pruim RJ, Welch RP, Sanna S, Teslovich TM, Chines PS, Gliedt TP, Boehnke M, Abecasis GR, Willer CJ: **LocusZoom: regional visualization of genome-wide association scan results**. *Bioinformatics* 2010, **26**(18):2336-2337.

2. Karolchik D, Baertsch R, Diekhans M, Furey TS, Hinrichs A, Lu Y, Roskin KM, Schwartz M, Sugnet CW, Thomas DJ: **The UCSC genome browser database**. *Nucleic acids research* 2003, **31**(1):51-54.
